# The Role of Hexon Amino Acid 188 Varies in Fowl Adenovirus Serotype 4 Strains with Different Virulence

**DOI:** 10.1128/spectrum.01493-22

**Published:** 2022-05-19

**Authors:** Baiyu Wang, Congcong Song, Panpan Yang, Mingzhen Song, Shiyi Zhao, Qilong Qiao, Zeng Wang, Jun Zhao

**Affiliations:** a College of Veterinary Medicine, Henan Agricultural University, Zhengzhou, China; University of Arizona

**Keywords:** fowl adenovirus serotype 4, hexon, virulence, pathogenesis, protein structure analysis

## Abstract

Hepatitis-hydropericardium syndrome (HHS) induced by fowl adenovirus serotype 4 (FAdV-4) has caused huge economic losses to poultry industries. The key genes responsible for different virulence of FAdV-4 strains are not fully elucidated. Previous studies indicated that hexon of pathogenic FAdV-4 has a conserved arginine (R) at position 188, and a conserved isoleucine (I) is present at this position in reported nonpathogenic FAdV-4. Recently, it was reported that R188 of hexon is the determinant site for pathogenicity of the emerging Chinese FAdV-4 strain. However, the role of hexon amino acid 188 (aa188) has not been examined in the nonpathogenic FAdV-4 strain. In this study, three recombinant FAdV-4 viruses, H/H/R188I, O/O/I188R, and H/O/I188R, were constructed by mutating hexon aa188 of FAdV-4 pathogenic strain CH/HNJZ/2015 (H) and nonpathogenic strain ON1 (O), and pathogenicity was assessed in specific-pathogen-free (SPF) chickens. Consistent with previous findings, H/O/I188R exhibited pathogenicity similar to that of CH/HNJZ/2015, yet H/H/R188I induced no mortality. Unexpectedly, all chickens infected with O/O/I188R survived. Postmortem examination of O/O/I188R-infected chickens showed typical lesions of inclusion body hepatitis rather than HHS. Expression of proinflammatory cytokines in CH/HNJZ/2015- and H/O/I188R-infected chickens was significantly higher than that in H/H/R188I-, ON1-, and O/O/I188R-infected chickens. Analysis of predicted hexon protein structures indicated that aa188 mutation leads to conformational changes in the L1 loop of HNJZ-hexon but not in ON1-hexon. In summary, the present study demonstrated that the role of hexon aa188 in the virulence of FAdV-4 varies between different strains. Induction of HHS requires factors aside from hexon aa188 in the emerging Chinese FAdV-4 strain.

**IMPORTANCE** HHS induced by FAdV-4 has caused huge economic losses to the poultry industry. The key determinants for the different virulence of FAdV-4 have not been fully elucidated. Here, we investigated the role of hexon aa188 in FAdV-4 strains with different virulence and showed that the role of hexon aa188 varies in FAdV-4 strains with different genetic contents. The hexon R188 may be the key amino acid for causing inclusion body hepatitis by the pathogenic FAdV-4 strain, and induction of HHS by FAdV-4 may need other viral cofactors. Moreover, the hexon R188I mutation greatly affected the expression of proinflammatory cytokines induced by the pathogenic strain CH/HNJZ/2015, but no significant difference was observed between the nonpathogenic strain ON1 and ON1 with hexon I188R mutation. We found that hexon aa188 mutation induced conformational changes to hexon protein in CH/HNJZ/2015 but not in ON1, which might be the underlying reason for the changing virulence.

## INTRODUCTION

Fowl adenoviruses (FAdV) of the genus *Aviadenovirus* and family *Adenoviridae* are categorized into five species designated FAdV-A to E and 12 serotypes, including FAdV-1 to 7, 8a, 8b, and 9 to 11. Hepatitis-hydropericardium syndrome (HHS) induced by FAdV-4 of FAdV-C and inclusion body hepatitis (IBH) induced by FAdV-8 of FAdV-E and FAdV-11 of FAdV-D are ubiquitous worldwide, causing significant economic losses to the world poultry industry (1–4). Up to 80% mortality was observed in chicken flocks infected with HHS, and necropsy indicated accumulation of pericardial effusion in the pericardial sac and necrotic foci and hemorrhages on the liver of infected chickens (5–7). IBH is associated with up to 30% mortality and is characterized by the presence of inclusion bodies in the hepatocytes of infected chickens (1, 8, 9).

FAdV-4 is a nonenveloped, double-stranded DNA virus with an approximately 43- to 45-kb genome encoding 11 structural proteins and 32 nonstructural proteins (10). Identification of key genes responsible for the pathogenicity of viruses is critical. Our previous works specifically revealed the genome differences in the fiber-1, fiber-2, penton, and hexon genes of FAdV-4 between the pathogenic and nonpathogenic strains (11). A 1,966-bp missing area between ORF42 and ORF43 was identified in the newly emerged pathogenic Chinese FAdV-4 strain; however, it is unrelated to the virulence of FAdV-4 (12, 13). Later, we demonstrated that both hexon and fiber-2 are closely associated with the virulence of FAdV-4, but fiber-1 and penton are irrelevant to the increased virulence of FAdV-4 in comparison to the nonpathogenic FAdV-4 strain (12, 14). Previous studies indicated that a pathogenic FAdV-4 strain with deletion of 7 to 40 amino acids (aa) in fiber-2 was highly attenuated (15). Fiber-1 of FAdV-4 is responsible for the attachment to the susceptible cells through the coxsackievirus and adenovirus receptor (16). However, one recent study showed that hexon, but not fiber-2, determines the virulence of the novel FAdV-4 strain and that the conserved arginine (R) at position 188 of hexon protein of pathogenic FAdV-4 strains plays a crucial role in the virulence of the Chinese FAdV-4 strain (17). These controversial results prompted us to explore the role of hexon aa188 in FAdV-4 strains with different virulence and genetic contents.

In the present study, the role of hexon aa188 was examined in both the pathogenic and nonpathogenic strains of FAdV-4. Hexon aa188 of the pathogenic strain and that of the nonpathogenic strain were substituted with one another by the FAdV-4 reverse genetic platform established previously (12). The virulence of the parental viruses and recombinant viruses was assessed in specific-pathogen-free (SPF) chickens.

## RESULTS

### Hexon aa188 mutation is irrelevant to th*e in vitro* replication of FAdV-4.

**FIG 1:**
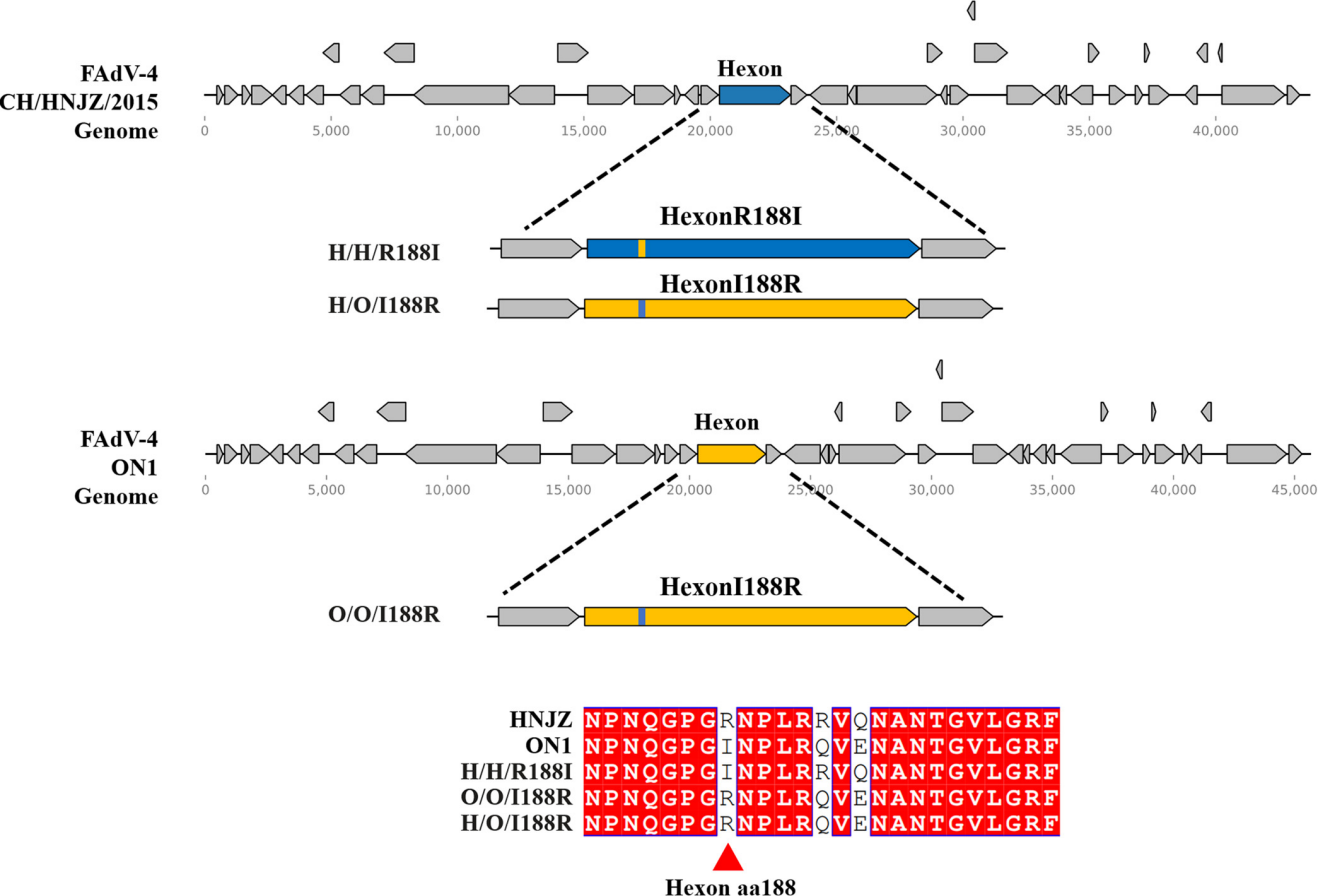
Generation of the recombinant viruses. The recombinant FAdV-4 viruses H/H/R188I and O/O/I188R were constructed by replacing the hexon aa188 of HNJZ with that of ON1 or vice versa; H/O/I188R was constructed by replacing the hexon of HNJZ with the hexon of ON1 with aa188 mutated back to that of HNJZ.

**FIG 2:**
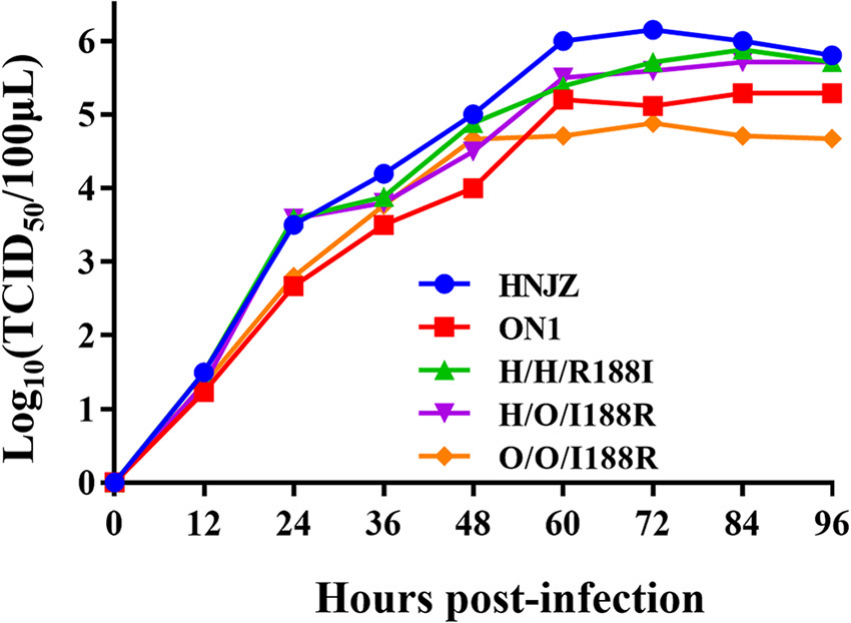
Growth curves of the recombinant viruses and parental viruses. LMH cells were infected with the recombinant FAdV-4 viruses H/H/R188I, O/O/I188R, and H/O/I188R and the parental viruses HNJZ and ON1 at an MOI of 0.001. The viruses were harvested at 0, 12, 24, 36, 48, 60, 72, 84, and 96 hpi for the median tissue culture infective dosage (TCID_50_) measurement.

To study the role of hexon aa188 in FAdV-4 with different virulence, recombinant viruses H/H/R188I, O/O/I188R, and H/O/I188R were constructed by a reverse genetic technique as illustrated in Fig. 1. The recombinant virus H/H/R188I was constructed by substitution of arginine at position 188 of hexon in the pathogenic strain with isoleucine at position 188 of hexon in the nonpathogenic strain, and O/O/I188R was constructed vice versa. The recombinant virus H/O/I188R was constructed using the previously constructed virus rHNJZ-hexon/ON1. The hexon of HNJZ was substituted by the hexon of ON1, and isoleucine at position 188 of ON1-hexon was mutated back to arginine. The recombinant viruses were rescued on Leghorn male hepatocellular (LMH) cells. The peak viral titers of HNJZ, ON1, H/H/R188I, O/O/I188R, and H/O/I188R were 10^6^, 10^5.3^, 10^5.7^, 10^4.9^, and 10^5.7^ median tissue culture infective dose (TCID_50_)/100 μL, respectively. The viral titers and *in vitro* replication capacity of the 3rd passage of the recombinant viruses were similar to those of the parental viruses on LMH cells (Fig. 2).

### The role of hexon aa188 varies in FAdV-4 strains with different virulence.

**FIG 3:**
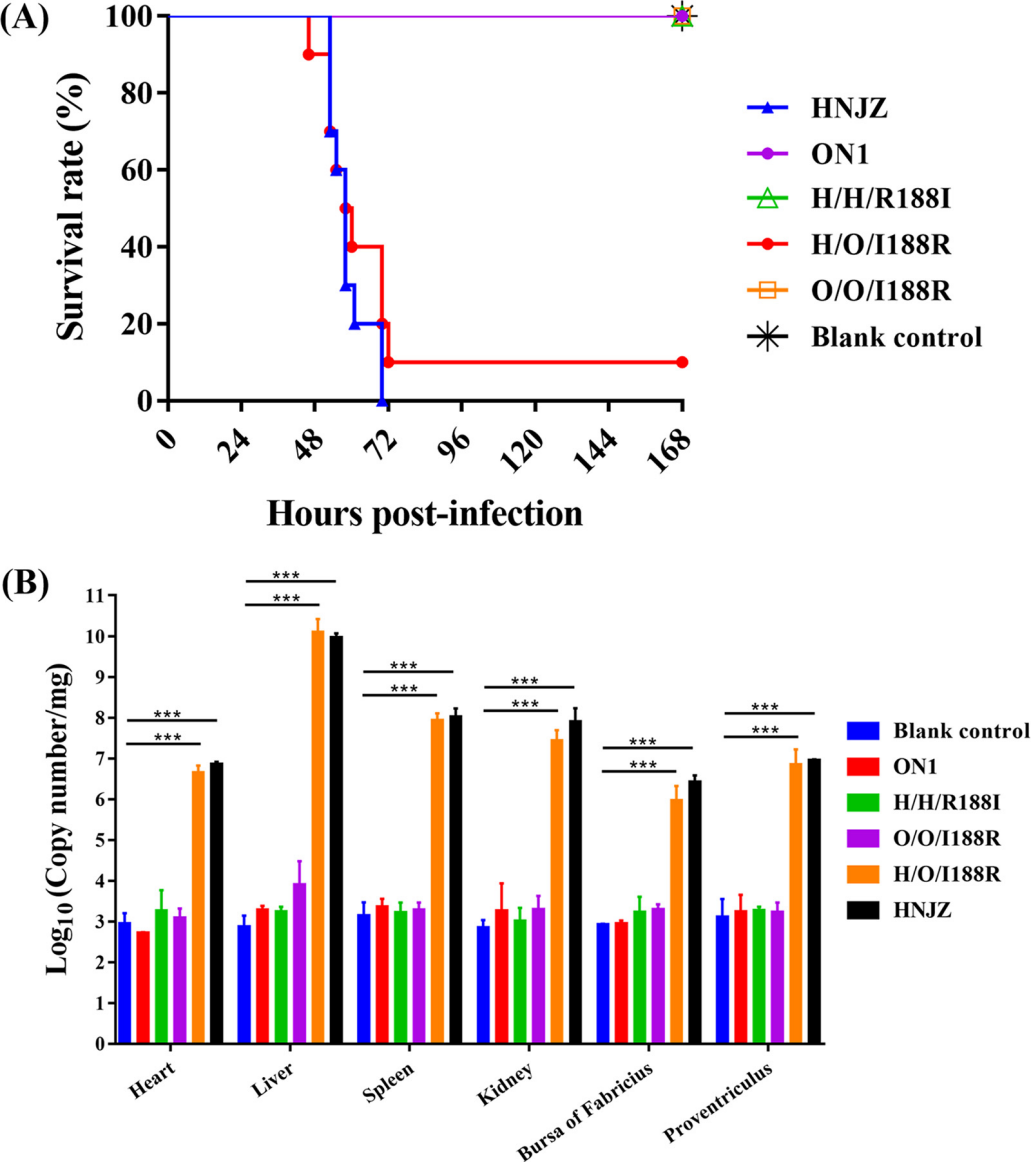
Pathogenicity test of HNJZ, ON1, H/H/R188I, O/O/I188R, and H/O/I188R. Three-week-old SPF chickens were inoculated intramuscularly with 0.2 mL of 2 × 10^5^ TCID_50_ of the recombinant viruses and the parental viruses and monitored for 7 days. The mortality was recorded, and tissue samples from the indicated organs were collected for viral load measurement. (A) The survival rates of SPF chickens after viral challenge. (B) The viral loads in different organs (***, *P* < 0.001).

The role of hexon aa188 in the pathogenicity of FAdV-4 was examined in SPF chickens. Three-week-old SPF chickens were inoculated with the same dosage of HNJZ, ON1, H/H/R188I, O/O/I188R, and H/O/I188R and monitored for 7 days. Chickens in the HNJZ and H/O/I188R groups exhibited typical clinical symptoms of HHS at 48 h postinfection (hpi), including severe depression, fluffy feathers, and diarrhea with green-yellowish excrement. Chickens in the H/H/R188I group showed milder depression and slightly reduced feed consumption at 48 hpi but recovered completely at 4 dpi, indicating that the hexon R188I mutation greatly reduced the virulence of HNJZ. Interestingly, chickens in the O/O/I188R group became depressed at 48 hpi with reduced feed intake and gradually recovered to normal at 6 dpi. Chickens in the ON1 and control groups showed no symptoms throughout the experiment. The mortality of chickens infected with HNJZ or H/O/I188R reached 100% at 70 hpi or 90% at 72 hpi, respectively. All chickens in the ON1, H/H/R188I, O/O/I188R, and control groups survived the experiment (Fig. 3A). However, it was observed that the size of chickens in the O/O/I188R group and H/H/R188I group was smaller than that in the ON1 and control groups due to the reduced feed consumption.

The viral copy numbers in the heart, liver, spleen, kidney, bursa of Fabricius, and proventriculus of the dead chickens and chickens euthanized at 7 dpi were determined. Only background levels of viral DNA were detected in the H/H/R188I, O/O/I188R, and control groups at 7 dpi. The viral loads detected in chickens in the H/H/R188I group were significantly lower than those in the HNJZ group, indicating that the hexon R188I mutation significantly suppressed the viral replication capacity of HNJZ in all tested viral targeting organs. However, the amount of viral DNA detected in the organs of chickens in the O/O/I188R group was at the same level as that in the ON1 group, suggesting that the hexon I188R mutation had almost no effect on the viral replication capacity of ON1 in the viral targeting organs (Fig. 3B). The effects of hexon aa188 mutation on the pathogenicity of FAdV-4 varies in FAdV-4 strains with different virulence and genetic contents.

**FIG 4:**
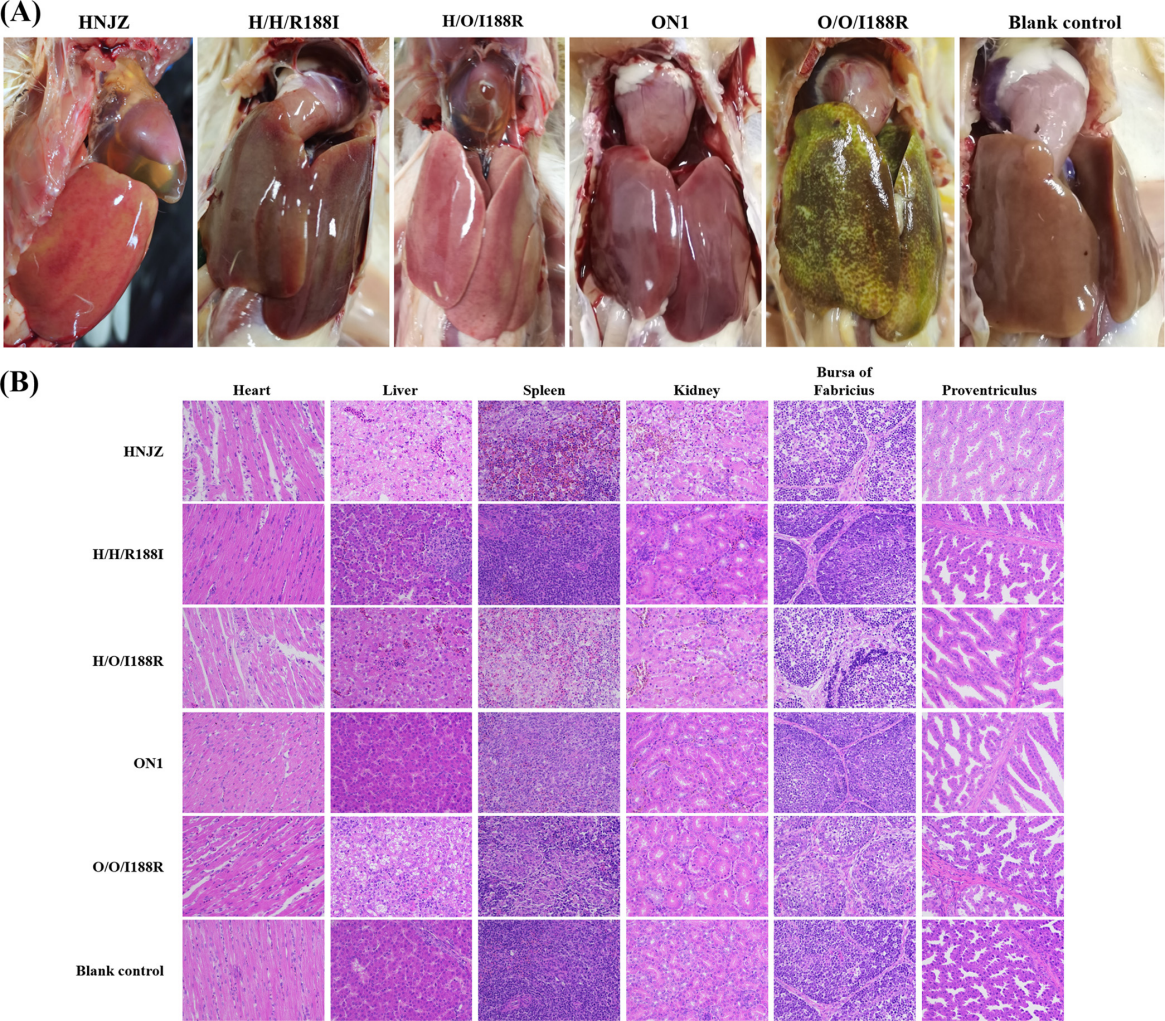
Presentative necropsy and histopathological analysis. (A) Pericardial effusion in the pericardial sac and swollen livers with focal hemorrhage were observed in the dead chickens infected with HNJZ and H/O/I188R. Typical appearance of IBH, including large areas of pale yellow-white discoloration and hemorrhages, was found on the livers of chickens infected with O/O/I188R. The livers of chickens infected with H/H/R188I and ON1 were highly similar to those of the control chickens. (B) Histopathological examination of the heart, liver, spleen, kidney, bursa of Fabricius, and proventriculus of chickens infected with HNJZ, ON1, H/H/R188I, O/O/I188R, and H/O/I188R and those of the control chickens (HE staining, original magnification ×400).

At necropsy, typical characteristics for HHS were observed in chickens infected with HNJZ and H/O/I188R, including accumulation of the clear, amber-colored liquid in the pericardial sac and swollen livers showing multifocal areas of necrosis (Fig. 4A). Histopathological analysis indicated myocarditis and granular degeneration in the heart, hepatocyte necrosis and lymphocytes infiltration in the liver, severe hemorrhage in the spleen, degeneration and enlargement of glomerulus with narrowed glomerular sacs and lymphocyte infiltration in the kidney, disintegration of lymphocytes in the bursa of Fabricius, and edema and necrosis in the proventriculus (Fig. 4B). Livers of chickens infected with O/O/I188R exhibited IBH indicative necropsy lesions. The liver was swollen and friable with large areas of pale yellow-white discoloration and pinpoint hemorrhages (Fig. 4A). It is worth noting, however, that there was no pericardial effusion in the pericardial sac of chickens infected with O/O/I188R. Histopathological lesions in chickens infected with O/O/I188R included myocarditis, vacuolar necrosis of hepatocytes and inclusion bodies in the hepatocytes, and depletion of lymphocytes in the spleen and the bursa of Fabricius. However, the kidneys and proventriculus had little difference compared to those of the control group. Collectively, these findings suggested that the hexon I188R mutation altered the virulence of ON1. Chickens in the ON1, H/H/R188I, and control groups euthanized at 7 dpi did not show any obvious lesions (Fig. 4B).

### The gene expression of the proinflammatory cytokines.

**FIG 5:**
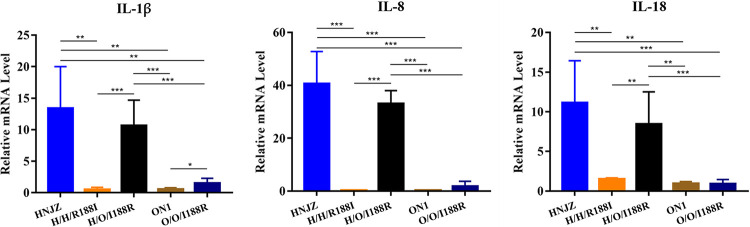
Expression levels of proinflammatory cytokine. The mRNA expression level of IL-1β, IL-8, and IL-18 in the liver of chickens infected with HNJZ, ON1, H/H/R188I, O/O/I188R, or H/O/I188R was measured by quantitative real-time RT-PCR (*, *P* < 0.05; **, *P* < 0.01; ***, *P* < 0.001).

To investigate the effect of hexon aa188 mutation on the expression of proinflammatory cytokines in chickens, total RNA was extracted from the liver tissues of chickens infected with HNJZ, ON1, H/H/R188I, O/O/I188R, and H/O/I188R for quantitative RT-PCR analysis. The results indicated that the expression levels of interleukin 1 beta (IL-1β), IL-8, and IL-18 were extremely significantly higher in the livers of HNJZ- and H/O/I188R-infected chickens than in those of the ON1-, H/H/R188I-, and O/O/I188R-infected chickens. Only the expression of IL-1β in the livers of O/O/I188R-infected chickens showed significant upregulation compared to that in those of the ON1-infected chickens, and the other two cytokines showed no significant changes (Fig. 5).

### Three-dimensional structural alignments of hexon of FAdV-4 with different virulence.

**FIG 6:**
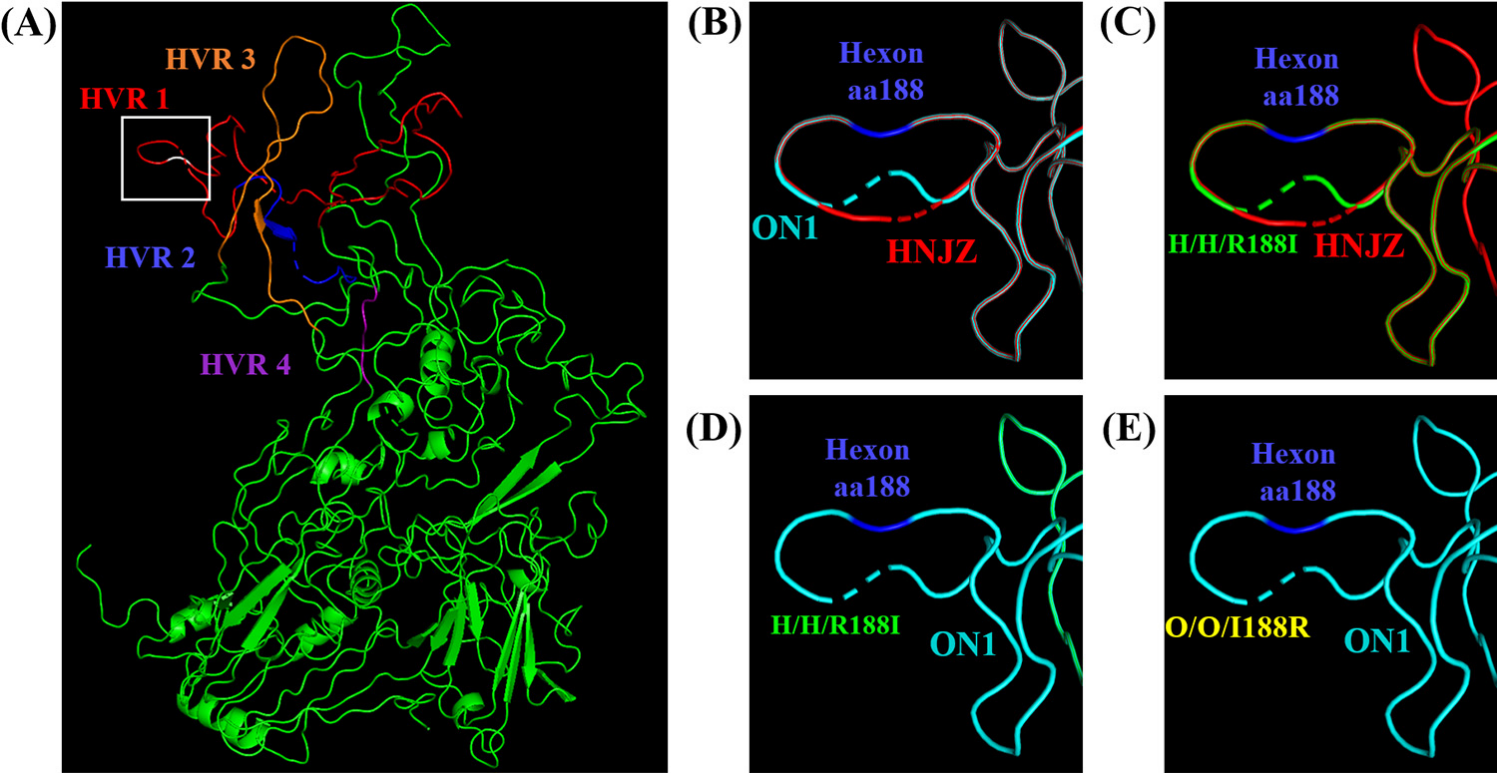
Predicted protein structure of hexon and conformational comparison at hexon aa188. The three-dimensional (3D) protein structures of hexon in different viruses were predicted by Phyre2 software and visualized in PyMol. (A) The predicted 3D structure of HNJZ-hexon protein with the hypervariable regions (HVRs) labeled (18). The aa188 of hexon in HVR1 is shown in panels B to E. (B) HNJZ-hexon versus ON1-hexon. (C) HNJZ-hexon versus H/H/R188I hexon. (D) ON1-hexon versus H/H/R188I hexon. (E) ON1-hexon versus O/O/I188R hexon.

To elucidate the reason that the role of hexon aa188 mutation varied in FAdV-4 with different virulence, we predicted the 3D protein structure of hexon with or without aa188 mutations by Phyre2 software and visualized it in PyMol (Fig. 6). The hypervariable regions (HVRs) in the L1 loop of hexon were labeled according to a previous study (18). The structures of HNJZ-hexon and ON1-hexon differ in the L1 loop at the highlighted region (Fig. 6B). The hexon R188I mutation in HNJZ leads to conformational changes (Fig. 6C), but no conformational change was predicted in O/O/I188R at the highlighted region (Fig. 6E). The H/H/R188I-hexon, O/O/I188R-hexon, and ON1-hexon are completely aligned at the highlighted region as illustrated in Fig. 6D and E.

## DISCUSSION

HHS caused by FAdV-4 has been spread worldwide after the first report in 1987 in Pakistan (19). The numerous outbreaks of HHS in China since 2015 have caused huge economic losses to the poultry industry due to the high mortality of the disease, ranging from 20% to 80% (20–23). To effectively control and prevent HHS, it is vital to understand the key determinants for the virulence of FAdV-4. The structural proteins fiber-1, fiber-2, penton, and hexon of FAdV-4 have become the major research interests to date. Our previous study identified a 1,966-bp genomic deletion in the Chinese epidemic strain HNJZ on the right-end region of the genome compared to the nonpathogenic strain ON1 and multiple amino acid mutations in the structural proteins (11). Later, we constructed recombinant FAdV-4 viruses HNJZ and ON1 containing fiber-1, fiber-2, penton, or hexon substituted by the counterparts and showed that the increased virulence of the Chinese FAdV-4 strain is closely related to fiber-2 and hexon but independent of fiber-1 and penton (12, 14). The 1,966-bp deletion was also irrelevant to the virulence of FAdV-4 as indicated by several studies (12, 13). Recently, Zhang et al. made several FAdV-4 recombinants with different residue mutations in hexon protein by using an emerging Chinese pathogenic FAdV-4 strain, HLJFAd15, as the backbone and compared the pathogenicity of these FAdV-4 recombinants with that of the wild-type strain. Their results showed that hexon, but not fiber-2, determines the virulence of the novel FAdV-4 strain and identified hexon residue 188 as the key amino acid for the virulence of the Chinese epidemic FAdV-4 strain (17). This result conflicts with previous research findings that both hexon and fiber-2 are closely associated with the virulence of the Chinese FAdV-4 strains, such as HNJZ and SD (12, 15, 24). Actually, the genome sequence identity between FAdV-4 strains HLJFAd15 (GenBank accession no. KU991797) and HNJZ (GenBank accession no. KU558760) is 100%. This prompted us to determine the role of hexon aa188 in FAdV-4 strains with different virulence and explore whether induction of HHS requires factors aside from the hexon aa188 in the pathogenic FAdV-4 strain.

To elucidate the role of hexon aa188 in FAdV-4 strains with different virulence, recombinant FAdV-4 viruses H/H/R188I, O/O/I188R, and H/O/I188R were constructed by using the FAdV-4 reverse genetic technique established previously (12). The hexon R188I mutation in HNJZ greatly reduced the virulence of the pathogenic Chinese epidemic FAdV-4 strain HNJZ: the mortality of chickens infected with H/H/R188I reduced to zero, and only a background level of viral loads was detected in the FAdV-4-targeting organs. Only slight depression and reduced feed consumptions were observed at 2 dpi and recovered at 4 dpi. The necropsy and histopathological analysis indicated no obvious lesions. Meanwhile, chickens infected with H/O/I188R showed clinical symptoms, necropsy and histopathological changes, tissue viral loads, and mortality similar to those of chickens infected with HNJZ. This result is consistent with the result of Zhang et al. indicating that the hexon residue 188 was the determinate factor for the virulence of the Chinese epidemic FAdV-4 strain (12). It should be pointed out, however, that there was no pericardial effusion in the pericardial sac of O/O/I188R-infected chickens, and O/O/I188R did not induce mortality, even though IBH indicative postmortem, and microscopic lesions were seen in livers of O/O/I188R-infected chickens. Our findings imply that the dominant role of hexon R188 in FAdV-4 virulence varies in FAdV-4 strains with different genetic contents, and the induction of HHS by FAdV-4 may need other viral cofactors. The hexon R188 may be the key amino acid for causing IBH by pathogenic FAdV-4, and the hexon aa188 mutation may alter hepatocyte tropism of FAdV-4 *in vivo*.

Pathogenic FAdV-4 infection induces significant upregulations of proinflammatory cytokines in the primary viral targeting organs such as the liver, spleen, and bursa of Fabricius (25–27). The changing virulence of FAdV-4 promoted us to study the inflammatory responses triggered by the parental FAdV-4 and the derivatives. The pattern recognition receptors, such as Toll-like receptors, form the first line of defense against invading pathogens (28). The downstream signaling of Toll-like receptors (TLRs) is conducted via MyD88 adaptors, which in turn activates NF-κB and the subsequent expression of downstream cytokines (29). IL-1β, IL-8, and IL-18 are important cytokines regulating the inflammatory responses at the site of infection (30, 31). Previous studies reported significant upregulations of IL-1β, IL-8, and IL-18 in the livers of pathogenic FAdV-4 infected chickens, which is consistent with our results (27, 32). Both our results and the study by Grgić et al. found no significant difference in the expression of IL-8 and IL-18 between the control and ON1-infected chickens (33). Interestingly, our results indicated that the hexon R188I mutation in HNJZ greatly reduced the expression of IL-1β, IL-8, and IL-18 compared to that in HNJZ in the infected livers; however, the hexon I188R mutation in ON1 showed little effect on the expression of inflammatory cytokines compared to ON1. Multiple studies indicated that excessive inflammatory responses might lead to organ injuries, especially heart injury and pericardial effusion, which might explain the acute death of HNJZ- and H/O/I188R-infected chickens (34). The large accumulation of pericardial effusion might be related to the excessive production of inflammatory cytokines (6), indicating that induction of the excessive inflammatory responses of the host might need other cofactors in the pathogenic FAdV-4 genome.

Hexon is one of the major structural proteins constituting the capsid of adenoviruses and playing an important role in the genome organization of adenoviruses (18). Most adenovirus structural or genomic studies were done with human adenovirus. Hexon consists of three identical monomers forming a pseudohexagonal shape (35). The external capsid side of the hexon monomer consists of loops L1, L2, and L4, which are responsible for the antibodies’ binding, and the internal capsid side consists of two conservative pedestal domains, P1 and P2, which participate in the hexon trimer formation. The L3 loop is buried internally and stabilizes the interface between the P1 and P2 conservative regions (35–37). Most amino acid variations, including mutations, deletions, and additions, are located at the HVRs in the three intertwined loops L1, L2, and L4. Of these, the L1 loop contains the highest variability, including HVR1 to 4, and hexon aa188 is located at the right end of HVR1 (18). Mutations in genes can affect the protein structure and, thus, the function of the protein. Hexon monomers of FAdV-4 comprise 937 aa which then form homotrimers. The hexon R188I mutation modifies the theoretical tertiary structure of HVR1 of HNJZ-hexon and is predicted to give it the same conformation as the ON1-hexon. This conformational change might influence the interaction of the pathogenic FAdV-4 strain HNJZ with the host factors through hexon, leading to a drastically reduced virulence. In contrast, the hexon I188R mutation of ON1-hexon has no effect on the theoretical tertiary structure of ON1-hexon. Although the hexon I188R mutation altered the pathogenicity of ON1, all chickens survived with background levels of viral loads detected in the primary viral targeting organs. Therefore, the alteration of hexon protein conformation might be the underlying cause of the changes in FAdV-4 virulence.

In summary, the present study showed that the role of hexon aa188 mutation varies in FAdV-4 strains with different virulence, and the induction of HHS by FAdV-4 might need a certain hexon conformation and participation of other viral cofactors.

## MATERIALS AND METHODS

### Cells and viruses.

The pathogenic FAdV-4 strain CH/HNJZ/2015, denoted as HNJZ (GenBank accession no. KU558760), was isolated from chickens suffering from HHS in Henan province, China as described previously (11). The nonpathogenic FAdV-4 strain ON1 (GenBank accession no. GU188428) was kindly provided by Éva Nagy from the Department of Pathobiology, University of Guelph, Guelph, Canada (38). Virus rescue, propagation, and *in vitro* characterization were performed using Leghorn male hepatocellular (LMH) cells (ATCC, CRL-2117) cultured in DME/F12 medium (Thermo Fisher Scientific, USA) supplemented with 10% fetal bone serum (FBS, AusgeneX, Australia) at 37°C with 5% CO_2_.

### Escherichia coli strains and plasmids.

Infectious clones of HNJZ and ON1, p15A-cm-HNJZ and p15A-cm-ON1, and the recombinant infectious clone p15A-cm-HNJZ-hexon/ON1 with HNJZ genome being the backbone but the hexon gene substituted by hexon of ON1 were constructed previously (12). The plasmid pR6K-amp-ccdB was used as a template to amplify the *ccdB* counter selectable cassette together with an ampicillin resistance gene. Recombinant plasmids pUC57-amp-HNJZ-hexonR188I and pUC57-amp-ON1-hexonI188R containing the hexon gene from HNJZ and ON1 with hexon aa188 substituted with the counterpart, respectively, were constructed in our lab. E. coli GBred-gyrA462 containing Redαβ recombinases and resistance to the *ccdB* toxin was used for substitution of the original hexon gene with the *amp-ccdB* cassette. E. coli GB05-dir, which is unresistant to *ccdB* toxin, was used for seamless substitution of the *amp-ccdB* cassette with the hexon mutants.

### Construction of recombinant FAdV-4 plasmids.

To examine the role of hexon aa188 in FAdV-4 strains with different pathogenicity, recombinant infectious clones of HNJZ and ON1 with mutations at position 188 of hexon protein were constructed. The original hexon gene in p15A-cm-HNJZ, p15A-cm-ON1, and p15A-cm-HNJZ-hexon/ON1 were replaced with the *amp-ccdB* counter selectable cassette by linear-circular homologous recombination (LCHR) in E. coli GBred-gyrA462 as described previously (12). The *amp-ccdB* cassettes for HNJZ or ON1 were amplified by PCR with specific oligonucleotides flanking with homologous arms and PacI digestion sites, as indicated in Table 1. The PCR was set up with 25 μL of the PrimeSTAR Max DNA polymerase (TaKaRa, cat. no. R045B), 1 μL (10 μM) of the forward and reverse primers, 2 μL of the template pR6K-amp-ccdB (100 ng), and 21 μL of ddH_2_O. The PCR protocol consisted of 30 cycles of 98°C for 10 s, 55°C for 10 s, and 72°C for 1 min. The amplicons were purified by gel electrophoresis and gel extraction. The infectious clones p15A-cm-HNJZ, p15A-cm-ON1, or p15A-cm-HNJZ-hexon/ON1, together with the purified *amp-ccdB* cassettes, were coelectroporated into E. coli GBred-gyrA462 for homologous recombination and selected by appropriate antibiotics as described in the previous protocol (39). The correct clones were named p15A-cm-HNJZ-hexon-amp-ccdB, p15A-cm-ON1-hexon-amp-ccdB, or p15A-cm-HNJZ-hexon/ON1-amp-ccdB. Next, the replacement of the *amp-ccdB* cassettes with the mutated hexon was performed in E. coli GB05-dir. The mutated hexon genes HNJZ-hexonR188I and ON1-hexonI188R were amplified using plasmids pUC57-amp-HNJZ-hexonR188I and pUC57-amp-ON1-hexonI188R as the templates and specific oligonucleotides with homolog arms as described in Table 1. The PCR and product purification were carried out as described above. The retrieved amplicons and the PacI-linearized p15A-cm-HNJZ-hexon-amp-ccdB, p15A-cm-ON1-hexon-amp-ccdB, or p15A-cm-HNJZ-hexon/ON1-amp-ccdB were treated with T4 DNA polymerase (NEB, USA, catalog no. M0203S) according to the previously described protocol and coelectroporated into E. coli GB05-dir for homologous recombination (40). The correct clones were verified by restriction enzyme digestions and sequencing and named p15A-cm-H/H/R188I, p15A-cm-O/O/I188R, and p15A-cm-H/O/I188R. The first letter indicated the virus backbone (i.e., HNJZ or ON1), the second letter indicated the source of hexon (i.e., HNJZ-hexon or ON1-hexon), and the third part indicated the mutation at position 188 of hexon (i.e., R188I indicated arginine mutated to isoleucine, and I188R indicated isoleucine mutated to arginine).

**TABLE 1:** Primers for construction of recombinant FAdV-4

Primer name	Sequence (5′–3′)^*a*^
HNJZ-hexon-ac-F	ACAACATCAATGTGGGCGACGGTTGGGTCCTGGACATGGGGTCGACCTAT**TTAATTAA**TTTGTTTATTTTTCTA
HNJZ-hexon-ac-R	AACCCGATGTAGTTGGGCCTGAGCGCACGCGTGGTGCCTATGTTATAATC**TTAATTAA**TTTGTTCAAAAAAAAG
ON1-hexon-ac-F	ACAACATCAATGTGGGCGACGGTTGGGTCCTGGACATGGGGTCGACCTAT**TTAATTAA**TTTGTTTATTTTTCTA
ON1-hexon-ac-R	AACCCGATGTAGTTGGGCCTGAGCGCACGCGTGGTGCCTATGTTATAATC**TTAATTAA**TTTGTTCAAAAAAAAG
HNJZ-hexon-F	ACAACATCAATGTGGGCGACGGTTGGGTCCTGGACATGGGGTCGACCTATTTCGACATCAAGGGAATCCTAGA
HNJZ-hexon-R	AACCCGATGTAGTTGGGCCTGAGCGCACGCGTGGTGCCTATGTTATAATCGTCGTAGTCCTCGGGAGGCGGCA
ON1-hexon-F	ACAACATCAATGTGGGCGACGGTTGGGTCCTGGACATGGGGTCGACCTATTTCGACATCAAGGGAATCCTAGA
ON1-hexon-R	AACCCGATGTAGTTGGGCCTGAGCGCACGCGTGGTGCCTATGTTATAATCGTCGTAATCCTCGGGAGGCGGCA

a Bold indicates restriction enzyme sites.

### Rescue of recombinant viruses.

The constructed infectious clones p15A-cm-H/H/R188I, p15A-cm-O/O/I188R, and p15A-cm-H/O/I188R were linearized with *Pme*I (NEB, USA, catalog no. R0560S) overnight at 37°C and purified by ethanol precipitation before transfection to LMH cells. LMH cells were seeded in T25 flasks a day before transfection. The transfection mixture was carried out with 5 μg of the linearized infectious clones and Lipofectamine 3000 according to the manufacturer’s instruction (Thermo Fisher Scientific, USA). The cells were harvested when obvious cytopathic effects occurred. The harvested viruses were centrifuged at 10,000 rpm for 1 min, and the supernatant was aliquoted and stored at −80°C. The viral DNA was extracted using TIANamp genomic DNA extraction kit (Tiangen, Beijing, China, catalog no. DP304) for sequencing identification. The successfully rescued recombinant viruses were named H/H/R188I, O/O/I188R, and H/O/I188R, respectively.

### *In vitro* characterization of recombinant viruses.

The viral titer and *in vitro* replication capacity of the 3rd passage of recombinant viruses were determined on LMH cells. LMH cell monolayers (10^5^ cells/well) were seeded in 96-well plates and inoculated with the parental viruses HNJZ and ON1 and the recombinant viruses H/H/R188I, O/O/I188R, and H/O/I188R, and each was serially diluted by 10-fold from 10^−1^ to 10^−10^ with 8 replicates. The cytopathic effects of LMH cells were observed under a light microscope at 96 hpi. The median tissue culture infective dose (TCID_50_) of the recombinant viruses was determined by the Reed-Muench method. The *in vitro* replication capacity of recombinant viruses was determined by the growth curve measurement. LMH cell monolayers (2 × 10^6^ cells/well) in 6-well plates were inoculated with HNJZ, ON1, H/H/R188I, O/O/I188R, or H/O/I188R at a multiplicity of infection (MOI) of 0.001. The viruses were harvested at 12, 24, 36, 48, 60, 72, 84, and 96 hpi for TCID_50_ measurement as described above.

### Pathogenicity study.

The pathogenicity of the rescued recombinant viruses was assessed in comparison with the parental viruses HNJZ and ON1 on 21-day-old SPF chickens (Beijing Boehringer Ingelheim Vital Biotechnology Co., Ltd., China). The animal study was conducted under the approval of the Animal Care and Use Committee of Henan Agricultural University (HNND2022030814). Sixty chickens were randomly divided into five infection groups, including H/H/R188I, O/O/I188R, H/O/I188R, HNJZ, and ON1, and one control group, with 10 chickens in each group. Chickens were separately housed in negative pressure isolators with sufficient supplies of food, water, and light. Chickens in the infection groups were inoculated intramuscularly with 0.2 mL of 2 × 10^5^ TCID_50_ of the corresponding virus and observed for 7 days. Tissue samples of the heart, liver, spleen, kidney, bursa of Fabricius, and proventriculus were collected from the dead chickens during the experiment and chickens euthanized at 7 dpi for viral load analysis, histopathological examination, and study of cytokine expression.

### Viral load measurement.

To determine the viral loads of viral targeting organs, total DNA was extracted from 100 mg of tissue samples of the heart, liver, spleen, kidney, bursa of Fabricius, and proventriculus with TIANamp genomic DNA extraction kit (Tiangen, Beijing, China, catalog no. DP304). A quantitative real-time PCR standard curve method for viral load measurement was established previously using the ORF14 gene of FAdV-4 as an indicator for the presence of HNJZ, ON1, and corresponding derivatives (12). Each reaction mixture was composed of 10 μL of 2× ChamQ Universal SYBR qPCR master mix (Vazyme, Nanjing, China), 0.5 μL of ORF14 forward and reverse primers (10 μM), and 2.5 μL of the extracted DNA in a total volume of 20 μL. The reaction cycles included one cycle at 95°C for 10 min and 40 cycles of 95°C for 10 s and 60°C for 1 min. The viral load was expressed as copy numbers per milligram of tissue samples.

### Histopathological examination.

To examine the microscopic lesions of viral targeting organs, the heart, liver, spleen, kidney, bursa of Fabricius, and proventriculus samples were placed in 10% formalin for fixation and embedded in paraffin. Sections of paraffin-embedded tissue samples were stained with hematoxylin and eosin following standard procedures. The stained samples were examined under light microscopy for the presence of lesions.

### Quantitative real-time RT-PCR analysis of proinflammatory cytokine expression.

To examine the expression levels of proinflammatory cytokines IL-1β, IL-8, and IL-18, the total RNA from 100 mg of liver tissue of chickens in each group was extracted using RNAsimple total RNA kit (Tiangen, Beijing, China, catalog no. DP419). Reverse transcription was performed with 2 μg RNA in a 20 μL reaction system using FastKing RT kit (Tiangen, Beijing, China, catalog no. KR116) according to the manufacturer’s instruction. The primers for detecting IL-1β, IL-8, and IL-18 were reported in previous studies (25, 33). The quantitative RT-PCR for each target gene was prepared in triplicates as follows: 10 μL of 2× ChamQ Universal SYBR qPCR master mix, 0.5 μL of each primer (10 μM), 2 μL of the cDNA, and 7 μL of double-distilled water (ddH_2_O). The reaction conditions were set as 1 cycle at 95°C for 30 s and then 40 cycles at 95°C for 10 s and 60°C for 30 s. GAPDH (glyceraldehyde-3-phosphate dehydrogenase) was used as the internal reference gene for the relative mRNA expression level analysis by the 2^−ΔΔ^*^Ct^* method.

### Hexon protein structure analysis.

The 3-dimensional structures of hexon of parental viruses HNJZ and ON1 and recombinant viruses H/H/R188I, O/O/I188R, and H/O/I188R were predicted with Phyre2 software and visualized in PyMol (http://www.pymol.org/) for structural comparison.

### Statistical analysis.

The statistical analysis was performed in GraphPad Prism 7 using the Student’s *t* test. The normality of data was confirmed by the D’Agostino-Pearson normality test. Parametric and unpaired *t* test was utilized to compare the viral loads and cytokine expressions between different groups. A *P* value smaller than 0.05 was considered to be significantly different.
